# Supplementary material for: Two-dimensional infrared-Raman spectroscopy as a probe of water’s tetrahedrality
Source: Nat Commun. 2023 Apr 7;14:1950. doi: 10.1038/s41467-023-37667-7 (PMC10082090; doi:10.1038/s41467-023-37667-7)
Supplement: Supplementary file 1 — Supplementary Information [file 41467_2023_37667_MOESM1_ESM.pdf]

# Supplementary information for: Two-dimensional infrared-Raman spectroscopy as a probe of water's tetrahedrality

Tomislav Begušić[1, *] and Geoffrey A. Blake[1, 2, *]

[1] *Division of Chemistry and Chemical Engineering, California Institute of Technology, Pasadena, California 91125, USA*

[2] *Division of Geological and Planetary Sciences, California Institute of Technology, Pasadena, California 91125, USA*

[*] Electronic mail: tbegusic@caltech.edu

[*] Electronic mail: gab@caltech.edu

## SUPPLEMENTARY DISCUSSION 1: TWO-DIMENSIONAL MODEL SYSTEM

Following Ito and Tanimura,[1] we simulated the spectra of two different two-dimensional models with harmonic Hamiltonian

$$H_0(q_1, q_2, p_1, p_2) = \sum_{i=1}^{2} \frac{p_i^2}{2} + \frac{1}{2}\omega_i^2 q_i^2, \tag{1}$$

$\omega_1 = 0.5$, $\omega_2 = 2$, and $\mu_1 = q_1$ and $\mu_2 = q_2$, where $\mu_1$ and $\mu_2$ are the dipole moments for the interaction with the first and second light pulses. For the model with mechanical anharmonicity, we set the Hamiltonian to

$$H(q_1, q_2, p_1, p_2) = H_0(q_1, q_2, p_1, p_2) + \sum_{i=1}^{2} \alpha_i q_i^4 + \lambda q_1 q_2^2, \tag{2}$$

where $\alpha_i = 2.5 \times 10^{-5} \omega_i^4$ and $\lambda = 0.1$, and we set $\Pi = q_2$. The weak quartic terms are added to ensure that the potential is bound. For the model with electrical anharmonicity, the Hamiltonian was quadratic, $H = H_0$, while $\Pi = -0.05 q_1 q_2$. The spectra (Supplementary Fig. 3) were simulated with exact quantum mechanics on a grid in position representation, similar to the benchmark simulations of Ref. 2. The response function was simulated for maximum $t_1$ and $t_2$ times of 50 with a time step of 0.25. Exponential damping $\exp(-(t_1 + t_2)/\tau)$ with $\tau = 1$ was applied to the response function before evaluating the discrete sine transform.

## SUPPLEMENTARY DISCUSSION 2: THEORY OF RII AND IIR RESPONSE FUNCTIONS

### A. Electrical anharmonic coupling

Here, we show that the nonlinear polarizability (for a RII pulse sequence) or dipole (for IIR) responsible for the first interaction with the external electric fields does not contribute to the response function. We must first assume that all types of anharmonicity are in the perturbative regime, i.e., that

$$R(t_1, t_2) \approx R^{\text{mech.}}(t_1, t_2) + R^{\text{elec.}}(t_1, t_2), \tag{3}$$

where $R^{\mathrm{mech.}}(t_1, t_2)$ is the term corresponding to the mechanical anharmonicity and considers only linear dipole and polarizability operators, while $R^{\mathrm{elec.}}(t_1, t_2)$ corresponds to the electrical anharmonicity and time evolution governed solely by the harmonic part of the Hamiltonian $H_0$. Terms that involve both types of anharmonicity simultaneously are neglected in this perturbative picture. Now let us consider the RII signal discussed in the main text, with $\Pi$ nonlinear in coordinates and $\mu(\mathbf{q}) = (\boldsymbol{\mu'})^T \cdot \mathbf{q}$ a linear function of coordinates. Then,

$$R^{\mathrm{RII,elec.}} = -\frac{1}{\hbar^2}\langle[[\hat{\mu}(t_2+t_1), \hat{\mu}(t_1)], \hat{\Pi}(0)]\rangle = 0 \tag{4}$$

because the commutator $[\hat{q}_i(t_1+t_2), \hat{q}_j(t_1)]$ is either zero (for $i \neq j$) or a time-dependent scalar (for $i == j$)[2] that then commutes with the polarizability operator. Therefore, the RII spectrum shown in Fig. 3 of the main text must be dominated by the mechanical anharmonic coupling mechanism.

## B. Comparing mechanical anharmonic coupling in IIR and RII

We now compare the RII spectrum to the IIR spectrum (which we call TIRV in the main text because there we focused on that specific frequency region). Only mechanical anharmonic coupling is considered, which means that the dipole and polarizability functions are assumed to be linear functions of coordinates. For IIR we have:

$$R^{\mathrm{IIR,mech.}}(t_1, t_2) = -\frac{1}{\hbar^2}\langle[[\hat{\Pi}(t_2+t_1), \hat{\mu}(t_1)], \hat{\mu}(0)]\rangle \tag{5}$$

$$= -\frac{1}{\hbar^2}\sum_{abc}\Pi'_c\mu'_b\mu'_a\langle[[\hat{q}_c(t_2+t_1), \hat{q}_b(t_1)], \hat{q}_a(0)]\rangle, \tag{6}$$

where the sum runs over all normal modes and $\mu'_a$ ($\Pi'_a$) is the derivative of the dipole moment (polarizability) with respect to mode $a$. Similarly, for RII,

$$R^{\mathrm{RII,mech.}}(t_1, t_2) = -\frac{1}{\hbar^2}\langle[[\hat{\mu}(t_2+t_1), \hat{\mu}(t_1)], \hat{\Pi}(0)]\rangle \tag{7}$$

$$= -\frac{1}{\hbar^2}\sum_{abc}\mu'_c\mu'_b\Pi'_a\langle[[\hat{q}_c(t_2+t_1), \hat{q}_b(t_1)], \hat{q}_a(0)]\rangle. \tag{8}$$

We see that the spectral feature due to the coupling between modes $a$ and $c$ is proportional to $\Pi'_c\mu'_a$ in IIR and $\mu'_c\Pi'_a$ in RII. Assuming that the one-dimensional IR and Raman spectra are dominantly harmonic, we have $I^{\mathrm{IR}}(\omega_a) \propto \mu'^2_a$ and $I^{\mathrm{Raman}}(\omega_a) \propto \Pi'^2_a$, where $\omega_a$ denotes

the frequency of mode $a$. Then, the IIR and RII spectra are related by

$$R^{\text{IIR,mech.}}(\omega_1, \omega_2) = g(\omega_1, \omega_2) R^{\text{RII,mech.}}(\omega_1, \omega_2) \tag{9}$$

$$g(\omega_1, \omega_2) = \sqrt{\frac{I^{\text{IR}}(\omega_1) I^{\text{Raman}}(\omega_2)}{I^{\text{Raman}}(\omega_1) I^{\text{IR}}(\omega_2)}}. \tag{10}$$

In the simulations presented in Fig. 3 of the main text, the RII spectrum was simulated using only the permanent part of the dipole moment, i.e., with $\boldsymbol{\mu}^{\text{ind}} = 0$. Therefore, Supplementary Eq. 10 was adjusted to

$$g(\omega_1, \omega_2) = \sqrt{\frac{I^{\text{IR}}(\omega_1) I^{\text{Raman}}(\omega_2)}{I^{\text{Raman}}(\omega_1) I^{\text{IR,perm}}(\omega_2)}}, \tag{11}$$

where $I^{\text{IR,perm}}(\omega)$ denotes the IR spectrum simulated with the permanent dipole moment. Also, because the IIR and RII response functions assumed $zz$-component of the polarizability tensor ($\Pi_{zz}$), the Raman spectrum in Supplementary Eq. (11) was computed as

$$I^{\text{Raman}}(\omega) = -\omega \text{Im} \int_0^\infty \langle \Pi_{zz}(t) \cdot \dot{\Pi}_{zz}(0) \rangle e^{-t^2/2\sigma_t^2} e^{-i\omega t} dt, \tag{12}$$

where $\sigma_t$ is defined in the Methods section of the main text. For completeness, the IR spectrum computed with full dipole, IR spectrum computed with permanent dipole only, and the $zzzz$ Raman spectrum are shown in Supplementary Fig. 4.

## SUPPLEMENTARY DISCUSSION 3: SPECTRUM DECOMPOSITION

Assuming sufficiently small $\varepsilon$, we can expand

$$\Pi(\mathbf{q}_{\pm,t}) = \Pi(\mathbf{q}_t) \pm \frac{\varepsilon}{2} \frac{d\Pi(\mathbf{q}_t)}{d\mathbf{p}_0} \cdot \frac{d\mu(\mathbf{q}_0)}{d\mathbf{q}_0} \tag{13}$$

$$= \Pi(\mathbf{q}_t) \pm \frac{\varepsilon}{2} \sum_{i=1}^{N_{\text{mol}}} \frac{d\Pi(\mathbf{q}_t)}{d\mathbf{p}_{i,0}} \cdot \frac{d\mu(\mathbf{q}_0)}{d\mathbf{q}_{i,0}} \tag{14}$$

to first order in $\varepsilon$, where the sum goes over $N_{\text{mol}}$ molecules, and $\mathbf{q}_i$ and $\mathbf{p}_i$ denote nine-dimensional position and momentum vectors of atomic coordinates in water molecule $i$. Then, it can be shown that

$$R^{\text{MD}}(t_1, t_2) = \sum_{i=1}^{N_{\text{mol}}} R_i^{\text{MD}}(t_1, t_2), \tag{15}$$

where

$$R_i^{\mathrm{MD}}(t_1, t_2) = \frac{\beta}{\varepsilon}\langle[\Pi(\mathbf{q}_{+,t_2}^{(i)}) - \Pi(\mathbf{q}_{-,t_2}^{(i)})]\dot{\mu}(\mathbf{q}_{-t_1})\rangle \tag{16}$$

and $\mathbf{q}_{\pm,t}^{(i)}$ is the position vector (all $3N_{\mathrm{atom}}$ coordinates) of a trajectory with initial momentum

$$\mathbf{p}_{\pm,0}^{(i)} = \mathbf{p}_0 \pm \frac{\varepsilon}{2}\begin{pmatrix} 0 \\ \vdots \\ \frac{d\mu(\mathbf{q}_0)}{d\mathbf{q}_{i,0}} \\ \vdots \\ 0 \end{pmatrix}, \tag{17}$$

i.e., a trajectory with the electric field applied only on molecule $i$. In our simulations, we did not decompose the spectrum into individual molecules, but into two groups of molecules exhibiting low and high tetrahedral order parameter, which was computed at time zero. Spectra evaluated in this way were divided by the fractions of molecules of a given order in the thermal distribution, which were $x(Q < 0.62) = \int_0^{0.62} P(Q)dQ = 0.388$ and $x(Q > 0.72) = \int_{0.72}^{1} P(Q)dQ = 0.403$ at 320 K. This temperature was chosen for the spectrum decomposition because it contains approximately equal number of molecules in the two groups. The tetrahedral order parameter was evaluated using the `order` code, which was obtained from https://github.com/ipudu/order.git.[3]

## SUPPLEMENTARY FIGURES

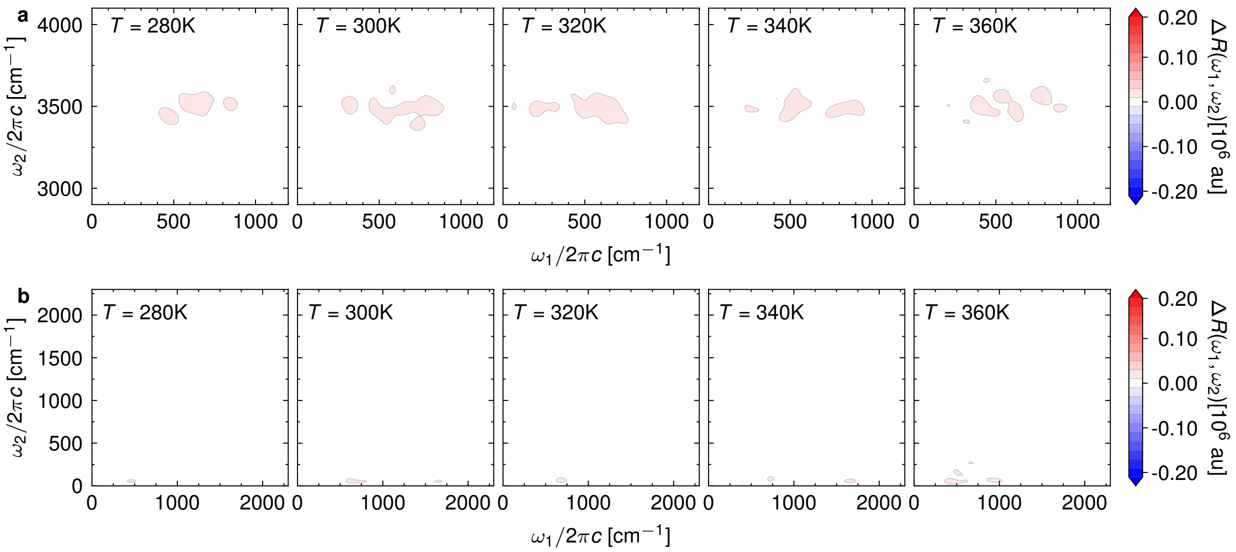


Supplementary Fig. 1. Statistical error. Statistical error of the IIR spectra computed with MD at different temperatures. **a** TIRV part of the spectrum. **b** Low-frequency part of the spectrum.

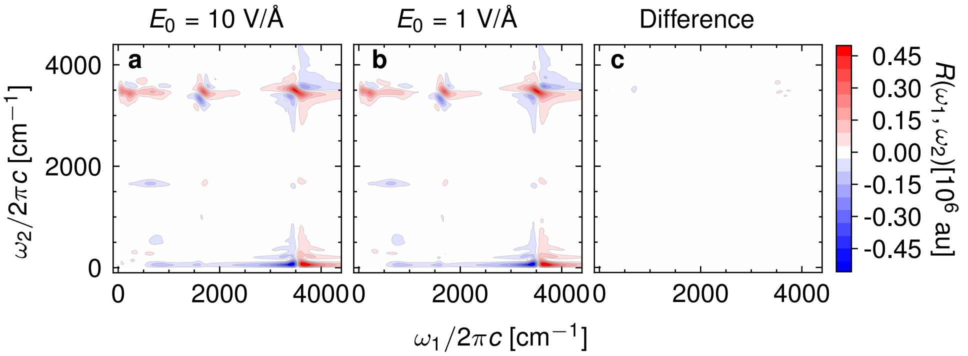


Supplementary Fig. 2. Electric field parameter. Difference (**c**) between IIR spectra simulated using MD at 280 K with $\varepsilon = 2$ ($E_0 = 10$ V/Å, **a**) and $\varepsilon = 0.2$ ($E_0 = 1$ V/Å, **b**). The difference is comparable to the statistical error presented in Supplementary Fig. 1.

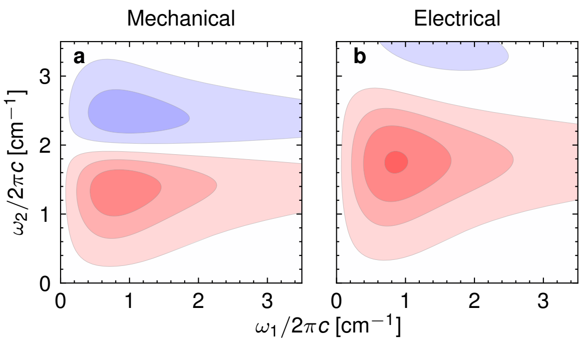


Supplementary Fig. 3. Two-dimensional model system. IIR spectra of the two-dimensional model systems (Supplementary Discussion 1) with mechanical (**a**) and electrical (**b**) anharmonic coupling.

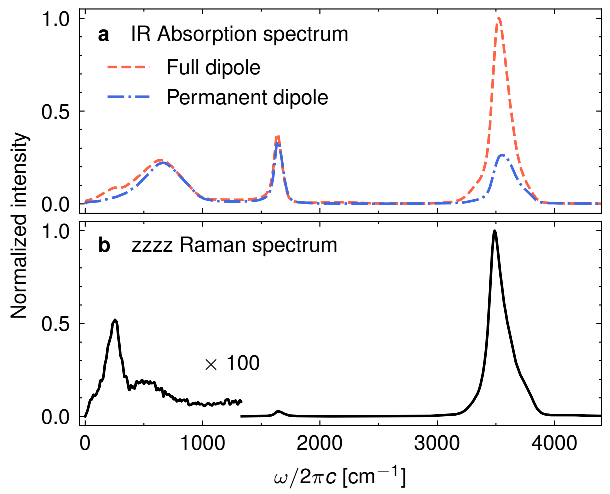


Supplementary Fig. 4. One-dimensional spectra used in Supplementary Eq. (11). **a** IR spectrum simulated according to Eq. (12) of the main text with full dipole moment (Eq. (10) of the main text) and permanent dipole moment ($\boldsymbol{\mu}^{\mathrm{ind}} = 0$ in Eq. (10) of the main text). **b** $zzzz$ Raman spectrum simulated according to Supplementary Eq. (12). All simulations were performed with MD at 300 K.

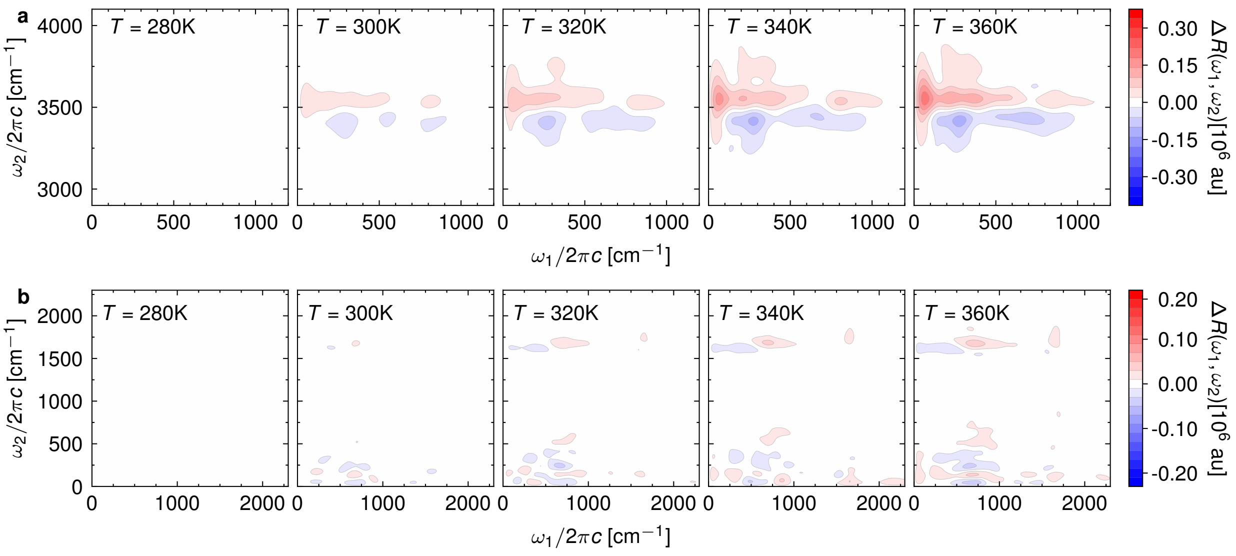


Supplementary Fig. 5. Difference IIR spectra. Difference between IIR spectra at different temperatures from the spectrum at 280 K. **a** TIRV part of the spectrum. **b** Low-frequency part of the spectrum.

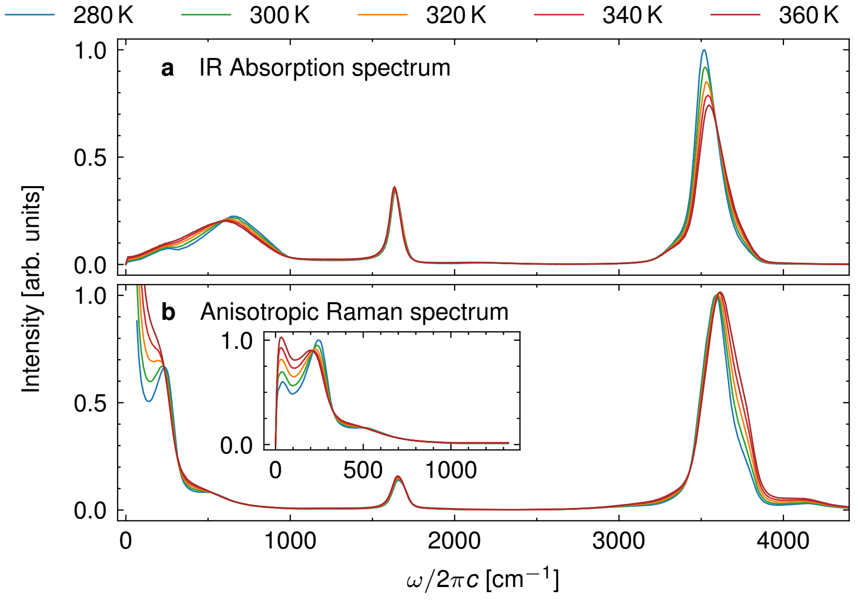


Supplementary Fig. 6. Temperature dependence of IR absorption (**a**) and anisotropic Raman (**b**) spectra of liquid water simulated with classical MD.

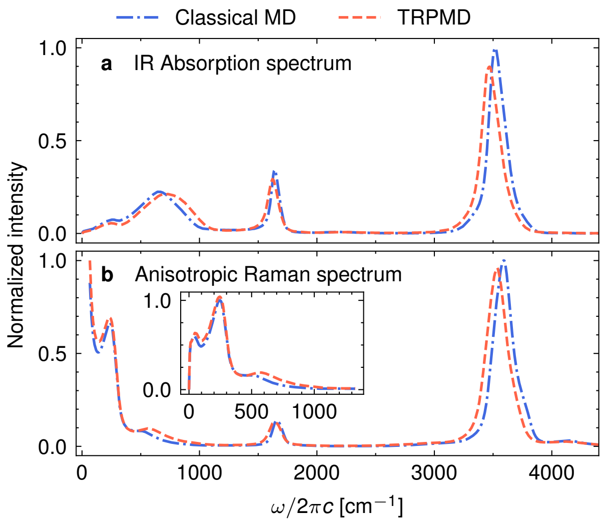


Supplementary Fig. 7. IR absorption (**a**) and anisotropic Raman (**b**) spectra of liquid water simulated with MD and TRPMD at 280 K, analogous to Fig. 1 of the manuscript. All spectra were scaled to the maximum intensity of the MD simulated spectrum.

## SUPPLEMENTARY REFERENCES

[1] H. Ito and Y. Tanimura, J. Chem. Phys. **144**, 074201 (2016).

[2] T. Begušić, X. Tao, G. A. Blake, and T. F. Miller, J. Chem. Phys. **156**, 131102 (2022).

[3] E. Duboué-Dijon and D. Laage, J. Phys. Chem. B **119**, 8406 (2015).
